# Association between vitamin D levels and preserved ratio impaired spirometry: an investigation of mediating roles of systemic inflammation and metabolic indicators

**DOI:** 10.3389/fnut.2025.1527333

**Published:** 2025-01-29

**Authors:** Tong Lin, Shanshan Huang, Fen Zhou, Xingkai Shen, Haiyan Mao

**Affiliations:** ^1^Department of Critical Care Medicine, Ningbo Medical Center Lihuili Hospital, Ningbo, China; ^2^Department of Geriatrics, Ningbo Medical Center Lihuili Hospital, Ningbo, China

**Keywords:** vitamin D, preserved ratio impaired spirometry, respiratory disease, mediation analysis, NHANES

## Abstract

**Background:**

Preserved ratio impaired spirometry (PRISm) represents an abnormal lung function state distinct from traditional chronic obstructive pulmonary disease, characterized by unique clinical and epidemiological features. PRISm has been associated with various health issues, including an increased risk of metabolic disorders and cardiovascular diseases. Vitamin D, known for its anti-inflammatory, immunomodulatory, and antioxidant properties, may play a role in reducing the risk of PRISm. This study aims to investigate the relationship between vitamin D levels and PRISm, including the mediating effects of systemic inflammation markers and metabolic indicators in a population of U.S. adults.

**Methods:**

This cross-sectional study analyzed data from 17,333 participants from the U.S. National Health and Nutrition Examination Survey, including 1,577 individuals with PRISm and 15,756 without. Baseline characteristics were assessed, and multivariate logistic regression models were employed to examine the relationship between vitamin D and PRISm. Mediation analysis was conducted to explore potential mediating roles of systemic immune-inflammation index (SII), triglyceride-glucose (TyG) index, and bilirubin. Nonlinear relationships were assessed using restricted cubic spline (RCS) models.

**Results:**

The PRISm group had lower median vitamin D levels and distinct inflammatory and metabolic profiles compared to the non-PRISm group. Multivariate analysis confirmed an inverse association between vitamin D levels and PRISm (adjusted OR: 0.989, 95% CI: 0.984–0.994, *p* < 0.001). RCS analysis showed a nonlinear protective effect of vitamin D, with risk stabilizing at levels above 50 nmol/mL. Mediation analysis highlighted bilirubin as a positive mediator (ACME = −4.11 × 10^−5^, *p* < 0.001), while TyG demonstrated a suppressive mediation effect (ACME = 2.68 × 10^−5^, *p* < 0.001). SII did not show significant mediation.

**Conclusion:**

Elevated vitamin D levels are linked to a lower risk of PRISm, with bilirubin potentially acting as a mediator in this protective relationship. This underscores the clinical significance of maintaining sufficient vitamin D levels to promote lung health and mitigate the prevalence of PRISm among U.S. adults. Further research is warranted to investigate personalized vitamin D supplementation strategies as a potential preventive approach.

## Introduction

Preserved ratio impaired spirometry (PRISm) is a condition in pulmonary function testing characterized by a preserved FEV1/FVC ratio (≥0.70) but reduced FEV1 (<80% of the predicted value), indicating impaired expiratory flow without meeting COPD criteria (1). Epidemiological studies of PRISm show that its prevalence in the general population ranges from approximately 10 to 12%, depending on the study population and specific definitions used (2–4). PRISm has been strongly associated with metabolic disorders and cardiovascular risk factors, such as obesity, insulin resistance, and chronic inflammation, with research indicating that individuals with PRISm are generally at an increased risk of all-cause mortality, particularly mortality related to cardiovascular conditions (5–7).

Vitamin D is a fat-soluble vitamin that is primarily synthesized in the skin through sunlight exposure, but it can also be obtained from certain foods (8). The relationship between vitamin D and respiratory diseases has become a growing area of research (9). Studies have shown that vitamin D is vital for regulating the immune system, controlling inflammatory responses, and maintaining lung function (10). Low levels of vitamin D are closely associated with the development and progression of various respiratory diseases, including asthma, chronic obstructive pulmonary disease (COPD), acute respiratory infections, and other pulmonary conditions (11–13). However, there is a lack of systematic research in the existing literature regarding the association between vitamin D and preserved ratio impaired spirometry (PRISm).

Therefore, we investigated the relationship between vitamin D and preserved ratio impaired spirometry (PRISm) using data from the National Health and Nutrition Examination Survey (NHANES). Chronic inflammation is a hallmark of impaired lung function and is associated with the pathogenesis of lung injury (14). The systemic immune inflammation index (SII), derived from neutrophils, lymphocytes, and platelet counts, is a robust biomarker of systemic inflammation. Vitamin D can modulate inflammatory pathways, and its deficiency is often linked to exacerbated inflammation (15). This makes SII an ideal biomarker for exploring the inflammatory mechanisms underlying the relationship between vitamin D and PRISm. The triglyceride-glucose (TyG) index is a reliable surrogate marker of insulin resistance (16). Insulin resistance exacerbates inflammation and oxidative stress, leading to decreased lung function (17), whereas vitamin D can improve insulin sensitivity and alleviate metabolic dysfunction (18). On the other hand, bilirubin, a potent endogenous antioxidant, neutralizes reactive oxygen species (ROS) and mitigates oxidative damage (19), a key mechanism of lung function decline and vitamin D metabolism (20). Elevated bilirubin levels are associated with improved lung function indicators (21), making it a valuable mediator for exploring the oxidative mechanisms between vitamin D and PRISm.

To investigate potential mechanisms, we considered the SII, TyG index, and bilirubin as mediators of inflammation, metabolic dysfunction, and oxidative stress, respectively. These mediators provide insights into the roles of inflammation, metabolism, and oxidative stress in the relationship between vitamin D and PRISm.

## Methods

### Study and data

**Figure 1:**
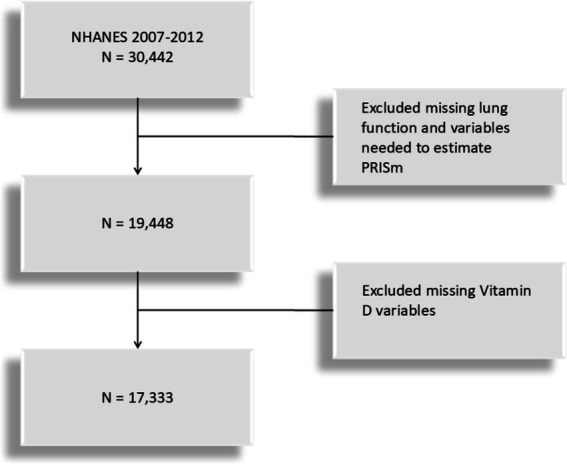
Flowchart for the study on preserved ratio impaired spirometry (PRISm) and vitamin D.

This study is based on data from the NHANES cross-sectional database, specifically covering lung function measurements from three NHANES cycles (2007–2008, 2009–2010, and 2011–2012). The study population includes U.S. adults aged 20 to 79 who meet the criteria for valid spirometry testing. Lung function, the primary variable, was extracted from NHANES examination data, while vitamin D levels were obtained from laboratory data. Participants with missing data on lung function or with missing variables for forced expiratory volume in one second (FEV1) was excluded and vitamin d was extracted through laboratory data. The participant selection flowchart is shown in Figure 1. The dataset is available at https://www.cdc.gov/nchs/nhanes/index.htm.

### Definitions of PRISm, TyG, and SII

Preserved ratio impaired spirometry (PRISm) was defined as a forced expiratory volume in one second/forced vital capacity ratio (FEV1/FVC) ≥0.7 with an abnormal spirometry result (FEV1 <80% of the predicted value) (22). Predicted FEV1 values were calculated using the Global Lung Function Initiative (GLI-2012) reference equations, implemented via specialized software available at https://gli-calculator.ersnet.org/index.html (23). Triglyceride-glucose (TyG) index is an indicator used to assess insulin resistance, calculated with the following formula: TyG index = Ln [fasting triglyceride (mg/dL) × fasting glucose (mg/dL)/2] (16). Systemic immune-inflammation index (SII) reflects an individual’s inflammatory state and is computed as: SII = Neutrophil count × Platelet count/Lymphocyte count.

### Covariates

Demographic information, including age, gender, ethnicity, and poverty income ratio (PIR), as well as health behaviors like smoking status and alcohol use, and medical history of cardiovascular disease and stroke, were gathered from NHANES using standardized questionnaires. Ethnicity was divided into categories: Mexican American, other Hispanic, non-Hispanic White, non-Hispanic Black, and other races. PIR was calculated as the ratio of family income to the federal poverty level per Department of Health and Human Services guidelines, with income levels classified as low (≤1.30), middle (1.31–3.50), and high (>3.50) (24). Body mass index (BMI) was computed as weight (kg) divided by height (m^2^) and grouped into normal weight (BMI <25 kg/m^2^), overweight (25 kg/m^2^ ≤BMI <30 kg/m^2^), and obese (BMI ≥30 kg/m^2^). Laboratory measures included cotinine, ALT, AST, bilirubin and creatinine, with eGFR calculated via the CKD-EPI equation (25). Cardiovascular disease and stroke were determined through affirmative answers to the question, “Has a doctor or health professional ever told you that you had congestive heart failure, coronary heart disease, angina, heart attack, or stroke?” Alcohol consumption was assessed by asking, “Have you had at least 12 alcoholic drinks in the past year?” Smoking status was evaluated based on responses to two questions: “Have you smoked at least 100 cigarettes in your lifetime?” and “Do you currently smoke?”

### Analysis of mediation

Parallel mediation analysis was conducted using the “Mediation” package in R software (version 4.0.0) with 1,000 bootstrap samples to evaluate the mediating roles of SII, TyG, and bilirubin in the relationship between vitamin D and PRISm risk. The direct effect represents the portion of vitamin D’s impact on PRISm that is not mediated by these factors, while the indirect effect reflects the portion mediated through them. The proportion of mediation was calculated as the ratio of the indirect effect to the total effect.

### Statistical analysis

We conducted weighted analyses following NHANES guidelines. Continuous variables that did not follow a normal distribution were presented as medians and interquartile ranges, and group comparisons were performed using the Mann–Whitney *U* test. Categorical variables were expressed as proportions, with group comparisons conducted via the *χ*^2^ test. For ordinal variables, proportions were used, and comparisons between groups were made with the Mann–Whitney *U* test. Multivariable logistic regression models were applied to examine the association between vitamin D and PRISm. Model 1 was unadjusted; Model 2 adjusted for sex, age, race, and PIR; and Model 3 adjusted for all covariates (sex, age, race, PIR, SII, TyG. bilirubin, BMI, cotinine, AST, ALT, and eGFR creatinine clearance, cardiovascular disease, stroke, alcohol consumption, and smoking status). Subgroup and interaction analyses were conducted to explore the relationship between vitamin D and PRISm across different population groups. To evaluate potential non-linear associations, we used restricted cubic spline analysis.

All statistical analyses were performed using R software (version 4.0.0) and SPSS (version 25.0), with statistical significance set at *p* < 0.05.

## Results

The baseline characteristics of the study participants, stratified by preserved ratio impaired spirometry (PRISm) status, revealed significant differences across multiple variables (Table 1). The total sample included 17,333 individuals, with 1,577 in the PRISm group and 15,756 in the non-PRISm group. Median vitamin D levels were lower in the PRISm group than in the non-PRISm group [53.10 (Q_1_, Q_3_: 37.30, 69.10) vs. 61.20 (Q_1_, Q_3_: 45.68, 77.00), *p* < 0.001]. Inflammatory markers such as the systemic immune-inflammation index (SII) were also lower in the PRISm group [409.09 (Q_1_, Q_3_: 278.85, 596.99) vs. 435.65 (Q_1_, Q_3_: 311.71, 619.03), *p* < 0.001], while the TyG index was slightly higher in the PRISm group [8.60 (Q_1_, Q_3_: 8.17, 9.20) vs. 8.53 (Q_1_, Q_3_: 8.06, 9.04), *p* < 0.001]. Bilirubin levels were significantly lower in the PRISm group [0.60 (Q_1_, Q_3_: 0.50, 0.80) vs. 0.70 (Q_1_, Q_3_: 0.60, 0.90), *p* < 0.001].

**Table 1:** The baseline characteristics of the study participants.

Variables	Total (*n* = 17,333)	Non-PRISm (*n* = 15,756)	PRISm (*n* = 1,577)	Statistic	*p*
Vitamin D (nmol/mL), M (Q₁, Q₃)	60.60 (44.90, 76.40)	61.20 (45.68, 77.00)	53.10 (37.30, 69.10)	−12.57	<0.001
SII, M (Q₁, Q₃)	433.06 (308.62, 617.60)	435.65 (311.71, 619.03)	409.09 (278.85, 596.99)	−5.03	<0.001
TyG, M (Q₁, Q₃)	8.54 (8.07, 9.05)	8.53 (8.06, 9.04)	8.60 (8.17, 9.20)	−4.36	<0.001
Bilirubin (mg/dL), M (Q₁, Q₃)	0.70 (0.60, 0.90)	0.70 (0.60, 0.90)	0.60 (0.50, 0.80)	−5.89	<0.001
Age, M (Q₁, Q₃)	39.00 (21.00, 54.00)	38.00 (20.00, 54.00)	41.00 (24.00, 56.00)	−3.25	0.001
Cotinine (ng/mL), M (Q₁, Q₃)	0.05 (0.02, 1.25)	0.05 (0.02, 1.21)	0.08 (0.02, 1.58)	−6.55	<0.001
eGFR (mL/min/1.73 m^2^), M (Q₁, Q₃)	92.88 (71.14, 112.56)	93.54 (71.72, 113.06)	87.23 (65.49, 107.28)	−7.24	<0.001
ALT (U/L), M (Q₁, Q₃)	20.00 (16.00, 27.00)	20.00 (16.00, 27.00)	20.00 (16.00, 27.00)	−0.38	0.707
AST (U/L), M (Q₁, Q₃)	23.00 (20.00, 28.00)	23.00 (20.00, 28.00)	23.00 (19.00, 28.00)	−1.41	0.158
Sex, *n* (%)				0.00	0.981
Male	8,776 (50.63)	7,978 (50.63)	798 (50.60)		
Female	8,557 (49.37)	7,778 (49.37)	779 (49.40)		
Race, *n* (%)				1134.74	<0.001
Mexican American	3,228 (18.62)	3,102 (19.69)	126 (7.99)		
Other Hispanic	1,938 (11.18)	1,824 (11.58)	114 (7.23)		
Non-Hispanic White	6,700 (38.65)	6,395 (40.59)	305 (19.34)		
Non-Hispanic Black	3,881 (22.39)	3,010 (19.10)	871 (55.23)		
Other race	1,586 (9.15)	1,425 (9.04)	161 (10.21)		
PIR, *n* (%)				−4.18	<0.001
≤1.3	5,787 (36.20)	5,224 (35.93)	563 (38.96)		
>1.3 and ≤3.5	5,682 (35.54)	5,124 (35.24)	558 (38.62)		
>3.5	4,517 (28.26)	4,193 (28.84)	324 (22.42)		
BMI (kg/m^2^), *n* (%)				−6.50	<0.001
<25	7,556 (43.82)	6,936 (44.21)	620 (39.95)		
25–30	4,743 (27.51)	4,414 (28.13)	329 (21.20)		
≥30	4,943 (28.67)	4,340 (27.66)	603 (38.85)		
Smoke, *n* (%)				0.01	0.908
Yes	5,366 (45.08)	4,871 (45.06)	495 (45.25)		
No	6,537 (54.92)	5,938 (54.94)	599 (54.75)		
Alcohol, *n* (%)				71.66	<0.001
Yes	8,444 (74.40)	7,788 (75.49)	656 (63.44)		
No	2,906 (25.60)	2,528 (24.51)	378 (36.56)		
Heart disease, *n* (%)				65.91	<0.001
No	11,235 (94.35)	10,261 (94.90)	974 (88.95)		
Yes	673 (5.65)	552 (5.10)	121 (11.05)		
Stroke, *n* (%)				24.17	<0.001
Yes	263 (2.21)	216 (2.00)	47 (4.29)		
No	11,634 (97.79)	10,586 (98.00)	1,048 (95.71)		
Diabetes, *n* (%)				183.32	<0.001
Yes	1,281 (7.40)	1,033 (6.56)	248 (15.75)		
No	15,796 (91.19)	14,501 (92.09)	1,295 (82.22)		
Borderline	245 (1.41)	213 (1.35)	32 (2.03)		

PRISm, preserved ratio impaired spirometry; SII, systemic immune-inflammation index; TyG, triglyceride-glucose index; BMI, body mass index; eGFR, estimated glomerular filtration rate; ALT, alanine aminotransferase; AST, aspartate aminotransferase.

The PRISm group had a slightly older median age [41.00 (Q_1_, Q_3_: 24.00, 56.00) vs. 38.00 (Q_1_, Q_3_: 20.00, 54.00), *p* = 0.001] and higher cotinine levels [0.08 (Q_1_, Q_3_: 0.02, 1.58) vs. 0.05 (Q_1_, Q_3_: 0.02, 1.21), *p* < 0.001]. Renal function, as measured by eGFR, was lower in the PRISm group [87.23 (Q_1_, Q_3_: 65.49, 107.28) vs. 93.54 (Q_1_, Q_3_: 71.72, 113.06), *p* < 0.001]. No significant difference in sex distribution or smoking status was observed between the groups. However, alcohol consumption patterns differed significantly, with a lower percentage of current drinkers in the PRISm group (63.44% vs. 75.49%, *p* < 0.001). Race and ethnicity varied, with a higher percentage of non-Hispanic Black individuals in the PRISm group (55.23% vs. 19.10%, *p* < 0.001). The PRISm group also had a higher prevalence of obesity (BMI ≥30) at 38.85%, compared to 27.66% in the non-PRISm group (*p* < 0.001). Comorbidities were more prevalent in the PRISm group, with higher rates of heart disease (11.05% vs. 5.10%, *p* < 0.001), stroke (4.29% vs. 2.00%, *p* < 0.001), and diabetes (15.75% vs. 6.56%, *p* < 0.001). Socioeconomic status, as indicated by the poverty-to-income ratio (PIR), also showed significant variation, with the PRISm group having a higher proportion in the lowest PIR category (≤1.3) compared to the non-PRISm group (38.96% vs. 35.93%, *p* < 0.001).

### Logistic regression analysis

In the multivariate logistic regression analysis examining the association between vitamin D and PRISM, three models were constructed with increasing adjustment for potential confounders (Table 2). In Model 1, without any adjustments, the odds ratio (OR) for vitamin D was 0.982 (95% CI: 0.978, 0.987; *p* < 0.001), indicating a significant inverse association between vitamin D and PRISM. Model 2 adjusted for sex, age, race, and poverty-income ratio (PIR), resulting in an OR of 0.987 (95% CI: 0.983, 0.991; *p* < 0.001), showing a slight attenuation in the inverse association. Model 3, further adjusted for SII, TyG, bilirubin, BMI, cotinine levels, AST, ALT, estimated glomerular filtration rate (eGFR), history of cardiovascular disease, stroke, alcohol consumption, smoking, and diabetes, yielded an OR of 0.989 (95% CI: 0.984, 0.994; *p* < 0.001). Although the strength of the association was further attenuated, the inverse relationship between vitamin D and PRISM remained statistically significant. Additionally, when categorized into quartiles, higher vitamin D levels (Q_1_–Q_4_) were consistently associated with lower odds of PRISM, with a statistically significant trend across all models (*p* for trend <0.001).

**Table 2:** Multivariate logistic regression analysis of the association between vitamin D and PRISm across different models.

	Model 1		Model 2		Model 3	
	OR (95% CI)	*p*	OR (95% CI)	*p*	OR (95% CI)	*p*
Vitamin D (nmol/mL)	0.982 (0.978, 0.987)	<0.001	0.987 (0.983, 0.991)	<0.001	0.989 (0.984, 0.994)	<0.001
Categories						
Q_1_ (<44.90)	Reference	/	Reference	/	Reference	/
Q_2_ (44.90–60.60)	0.571 (0.48, 0.68)	<0.001	0.699 (0.583, 0.838)	<0.001	0.674 (0.533, 0.851)	0.002
Q_3_ (60.60–76.40)	0.409 (0.333, 0.502)	<0.001	0.514 (0.421, 0.628)	<0.001	0.519 (0.383, 0.705)	<0.001
Q_4_ (>76.40)	0.324 (0.253, 0.414)	<0.001	0.434 (0.34, 0.554)	<0.001	0.478 (0.358, 0.636)	<0.001
*p* for trend	/	<0.001	/	<0.001	/	<0.001

Model 1: unadjusted; Model 2: adjusted for gender, age, ethnicity, and poverty income ratio; Model 3: adjusted for all covariates (gender, age, ethnicity, PIR, SII, TyG, bilirubin, BMI, cotinine, AST, ALT, GFR creatinine clearance, cardiovascular disease, stroke, alcohol consumption, and smoking status); PRISm, preserved ratio impaired spirometry; OR, odds ratio; CI, confidence interval.

### Nonlinear analysis

**Figure 2:**
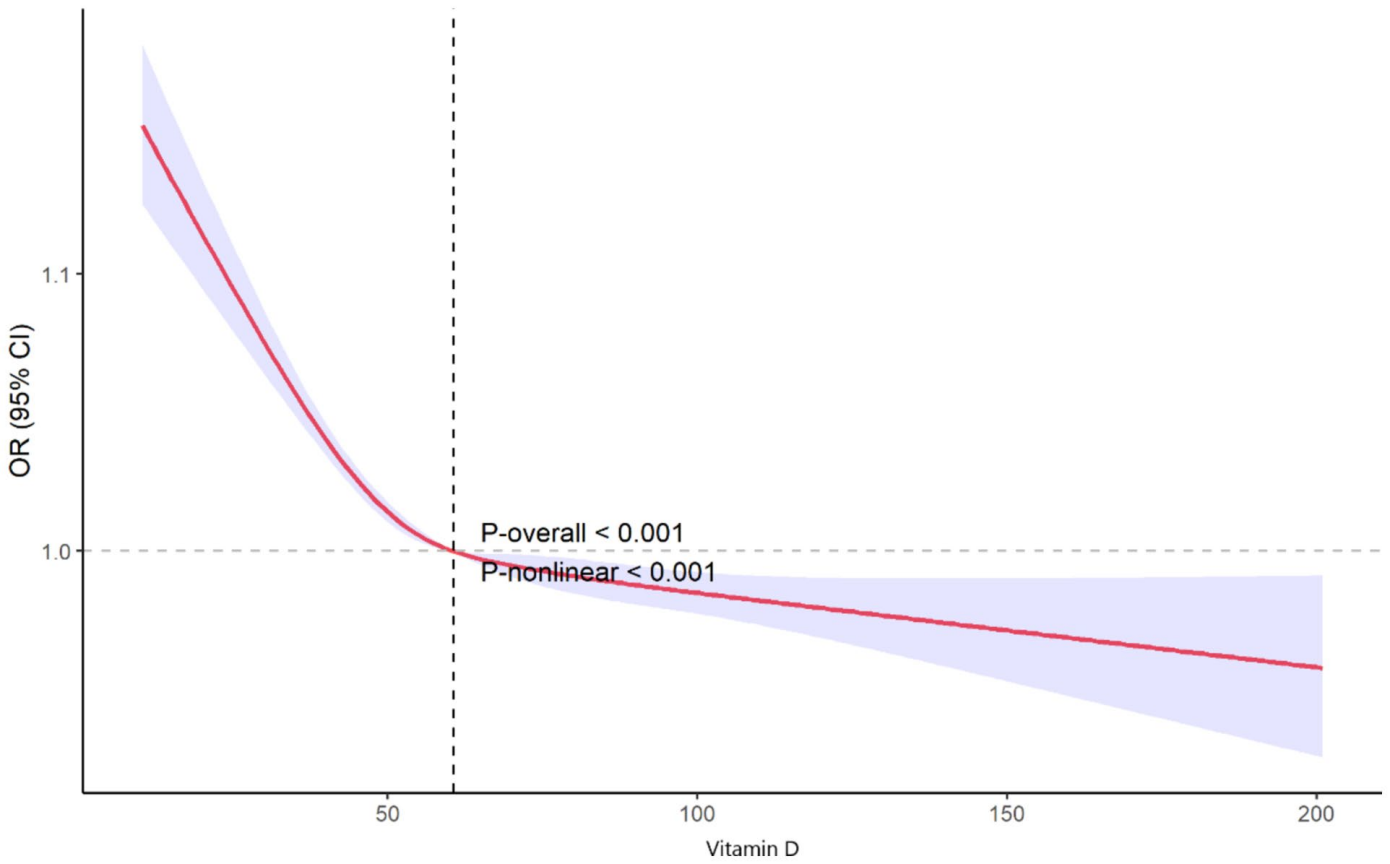
Nonlinear association between vitamin D levels and odds of preserved ratio impaired spirometry.

The restricted cubic spline (RCS) plot illustrates the nonlinear association between vitamin D levels and the odds ratio (OR) of PRISm (Figure 2). As shown in the graph, lower vitamin D levels are associated with a higher risk of PRISm. The curve demonstrates a sharp decline in the odds ratio as vitamin D levels increase from very low values, approaching an OR of 1.0 around a vitamin D level of 50 nmol/L. Beyond this level, the association continues to decline, stabilizing near an OR of 1.0 around 60.6 nmol/L (dashed vertical line). This indicates that higher vitamin D levels are associated with a stable or slightly reduced risk of PRISm. Both the overall trend and the nonlinear relationship are statistically significant (*p*-overall <0.001 and *p*-nonlinear <0.001).

### Subgroup and interaction analysis

The subgroup analysis shows that the association between vitamin D levels and the odds of PRISm varies slightly across different demographic and socioeconomic groups. Overall, the odds ratio (OR) for PRISm per unit increase in vitamin D is 0.989 (95% CI: 0.984, 0.994, *p* < 0.001), indicating a protective effect of higher vitamin D levels.

When analyzed by gender, the effect was significant for both males (OR = 0.988, 95% CI: 0.980, 0.996, *p* = 0.004) and females (OR = 0.990, 95% CI: 0.985, 0.995, *p* < 0.001). Age stratification showed significant effects in both age groups: individuals younger than 40 years had an OR of 0.985 (95% CI: 0.974, 0.996, *p* = 0.010), while those 40 years or older had an OR of 0.990 (95% CI: 0.983, 0.997, *p* = 0.004). Race and ethnicity groups exhibited varying levels of significance. While most groups did not reach statistical significance, non-Hispanic Black participants showed a borderline association (OR = 0.994, 95% CI: 0.988, 1.000, *p* = 0.054). In terms of poverty-to-income ratio (PIR), the association remained significant across all subgroups, with stronger effects observed in participants with a PIR between 1.3 and 3.5 (OR = 0.986, 95% CI: 0.978, 0.993, *p* = 0.001) and a weaker effect in those with PIR >3.5 (OR = 0.993, 95% CI: 0.986, 1.000, *p* = 0.041). The interaction *p*-values suggest no significant effect modification by gender (*p* = 0.434), age (*p* = 0.771), race/ethnicity (*p* = 0.744), or PIR (*p* = 0.854), indicating that the association between vitamin D levels and PRISm is consistent across these subgroups (Table 3).

**Table 3:** Subgroup analysis of the association between vitamin D levels and odds of preserved ratio impaired spirometry.

Subgroup	OR (95% CI)	*p*	*p* for interaction
Overall	0.989 (0.984, 0.994)	<0.001	
Gender			0.434
Male	0.988 (0.980, 0.996)	0.004	
Female	0.990 (0.985, 0.995)	<0.001	
Age			0.771
<40	0.985 (0.974, 0.996)	0.010	
≥40	0.990 (0.983, 0.997)	0.004	
Race			0.744
Mexican American	0.983 (0.961, 1.004)	0.114	
Other Hispanic	0.999 (0.983, 1.015)	0.902	
Non-Hispanic White	0.998 (0.990, 1.006)	0.671	
Non-Hispanic Black	0.994 (0.988, 1.000)	0.054	
Other race	0.998 (0.983, 1.013)	0.774	
PIR			0.854
≤1.3	0.990 (0.982, 0.998)	0.013	
>1.3 and ≤3.5	0.986 (0.978, 0.993)	0.001	
>3.5	0.993 (0.986, 1.000)	0.041	

### Mediation analysis

**Figure 3:**
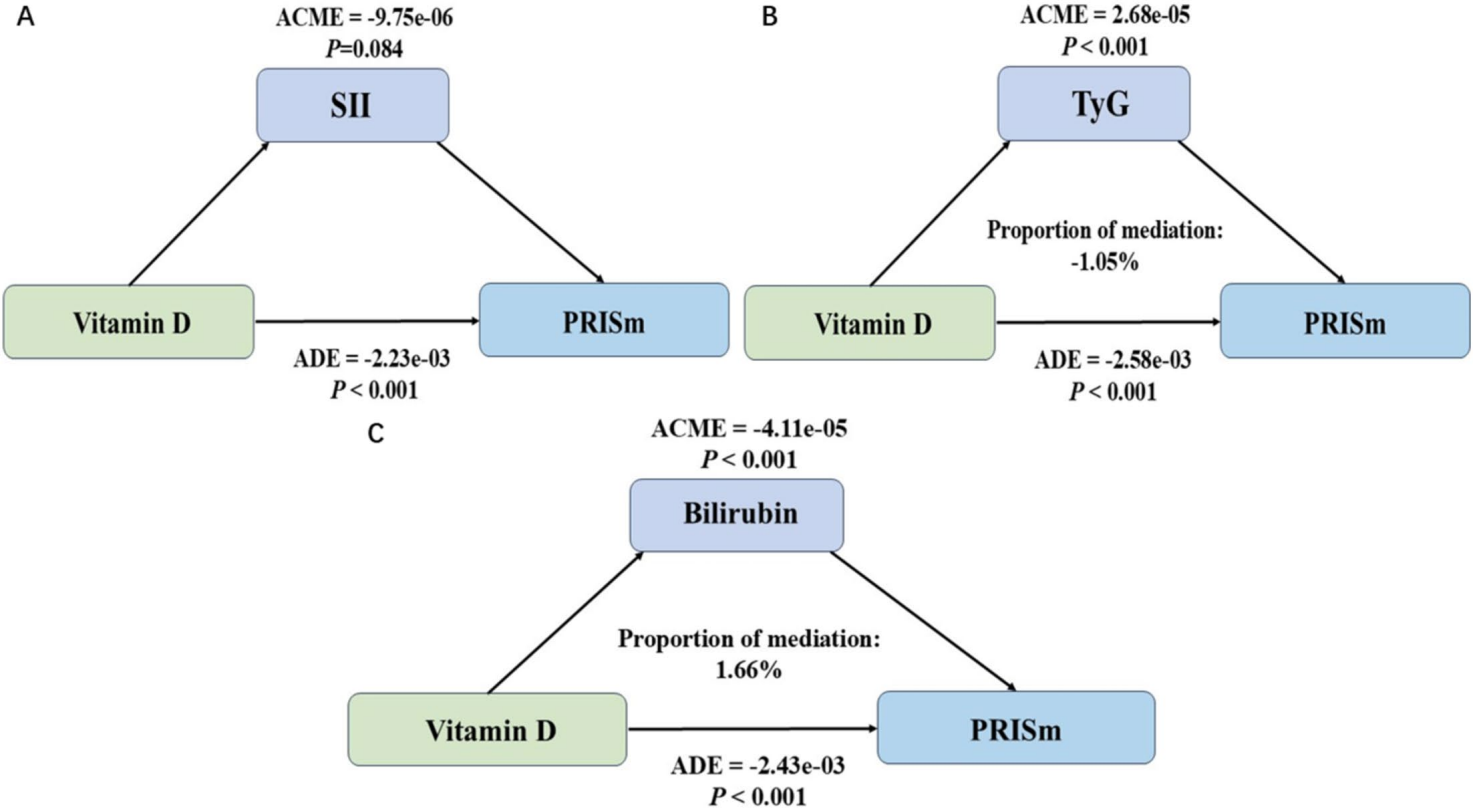
Mediation analysis of the association between vitamin D levels and preserved ratio impaired spirometry (PRISm) using systemic inflammatory index (SII), triglyceride-glucose index (TyG), and bilirubin as mediators. **(A)** Mediation analysis using systemic inflammatory index (SII) as the mediator. **(B)** Mediation analysis using triglyceride-glucose index (TyG) as the mediator. **(C)** Mediation analysis using bilirubin as the mediator (ACME, average causal mediation effect; ADE, average direct effect).

The mediation analysis revealed distinct roles of SII, TyG, and bilirubin in the association between vitamin D levels and PRISm (Figure 3). Figure 3A indicated that SII did not serve as a significant mediator (ACME = −9.75 × 10^−6^, *p* = 0.084), suggesting that systemic inflammation, as represented by SII, does not contribute to the mediating pathway between vitamin D and PRISm. In contrast, Figure 3B showed that TyG had a statistically significant mediating effect (ACME = 2.68 × 10^−5^, *p* < 0.001), but the proportion of mediation was negative (−1.05%), indicating that while the pathway is significant, its contribution may have an inverse or suppressive influence in the overall association. This negative mediation proportion suggests complexities in the metabolic pathways linking vitamin D to lung function. Figure 3C demonstrated that bilirubin acted as a significant mediator (ACME = −4.11 × 10^−5^, *p* < 0.001), with a positive mediation proportion of 1.66%, underscoring its potential role in mediating the protective effects of vitamin D on PRISm.

## Discussion

Preserved ratio impaired spirometry (PRISm) is receiving increasing attention due to its high prevalence and strong association with disease progression. Studies indicate that 22.2 to 35.8% of individuals with PRISm are expected to develop COPD within 5 years, highlighting its importance as a target for early intervention (4). Additionally, PRISm is associated with multiple health conditions, including cardiovascular disease, metabolic syndrome, and increased all-cause mortality, underscoring its complex impact on overall health and prognostic value (5–7). Therefore, early identification and management of PRISm are essential for preventing disease progression and improving patient outcomes. Vitamin D is an essential fat-soluble vitamin that plays a critical role in regulating calcium and phosphorus metabolism, maintaining bone health, and supporting various physiological functions. Its anti-inflammatory effects are achieved through the regulation of immune cell activity and cytokine secretion, linking it closely to respiratory diseases such as COPD and respiratory infections (9, 11–13).

The results of this study demonstrate a significant inverse association between vitamin D levels and PRISm. Vitamin D levels in the PRISm group were markedly lower than those in the non-PRISm group. In the unadjusted model, each unit increase in vitamin D was significantly associated with a reduced risk of PRISm. Although this association was somewhat attenuated after adjusting for sex, age, race, and socioeconomic factors (such as poverty-to-income ratio), it remained statistically significant. In the fully adjusted model, higher vitamin D levels continued to be associated with a lower risk of PRISm. These findings suggest that vitamin D deficiency may be an important risk factor for impaired lung function. Vitamin D exerts its anti-inflammatory effects by regulating immune cell activity and cytokine secretion, inhibiting the release of pro-inflammatory cytokines (e.g., IL-6, TNF-α, IL-2), and promoting the production of anti-inflammatory cytokines (e.g., IL-4, IL-5, IL-10) (10, 26). This regulation helps reduce chronic inflammation-related damage to lung tissue, thereby protecting lung function. By binding to the vitamin D receptor (VDR) within the immune system, vitamin D modulates the activity of T cells and macrophages, balancing the Th1/Th2 cell response and preventing tissue damage caused by excessive immune responses and inflammatory cytokine release (27). The absence of VDR in airway epithelial cells can lead to a loss of epithelial integrity (28). Additionally, vitamin D mitigates lung injury by stimulating epithelial repair, reducing epithelial cell apoptosis, and inhibiting TGF-β-induced epithelial-to-mesenchymal transition (29).

In the subgroup analysis, the association between vitamin D levels and PRISm in non-Hispanic Black participants showed borderline significance (*p* = 0.054), potentially indicating a weaker or less consistent relationship compared to other subgroups. Studies have shown that vitamin D deficiency is more prevalent among non-Hispanic Black individuals, with 11.9% having levels below 25 nmol/L, a proportion significantly higher than in other racial groups (30). Potential racial differences in vitamin D metabolism, such as genetic variations affecting its bioavailability and the presence of melanin in darker skin reducing ultraviolet penetration and subsequent vitamin D3 synthesis, may contribute to lower 25(OH)D levels (31, 32). Further investigations into targeted interventions or the unique risk profiles of non-Hispanic Black individuals may help clarify the observed trends.

In the mediation analysis, bilirubin emerged as a positive mediator in the relationship between vitamin D and PRISm. Bilirubin is widely recognized for its antioxidant properties, which enable it to neutralize reactive oxygen species (ROS) and mitigate oxidative stress (33). Studies have shown that bilirubin levels are negatively correlated with the severity and progression of COPD (34). Oxidative stress and oxidative damage play critical roles in the pathogenesis of COPD, where oxidative stress products damage cellular DNA, lipids, carbohydrates, and proteins, initiating a series of pathological processes that contribute to the onset and progression of COPD (35). Bilirubin, with its antioxidant and anti-inflammatory properties, can protect lung tissue by scavenging reactive oxygen species (ROS) and alleviating oxidative stress (34, 36). Therefore, the potential clinical importance of maintaining adequate bilirubin levels as part of an antioxidative defense mechanism in preventing or managing PRISm. In contrast, the TyG index, a reliable marker of insulin resistance, exhibited a suppressive effect in the mediation analysis. This suggests that insulin resistance, reflected by elevated TyG levels, may counteract some of the protective effects of vitamin D on PRISm. Insulin resistance is known to exacerbate systemic inflammation and oxidative stress, both of which are detrimental to lung function (17). However, vitamin D plays a crucial role in modulating these metabolic pathways by improving insulin sensitivity and reducing chronic inflammation (15, 18). Therefore, despite the suppressive effect of TyG, vitamin D exerts an overall beneficial influence on metabolic health, which contributes to lowering the risk of PRISm. These findings highlight the complex interplay between vitamin D, oxidative stress, and metabolic dysfunction, offering insights into potential therapeutic strategies targeting these pathways.

Compared to traditional linear or polynomial models, restricted cubic spline (RCS) analysis offers greater precision in detecting thresholds and inflection points, enabling a clearer understanding of dose–response relationships (37). Non-linear analysis using RCS curves revealed a significant increase in PRISm risk at lower vitamin D levels. As vitamin D levels reached approximately 50 nmol/L, the risk declined sharply and began to stabilize, with the odds ratio approaching 1.0. Beyond this point, the association continued to flatten, stabilizing further around 60.6 nmol/L (dashed vertical line), which represents the level at which the risk of PRISm is minimized. Based on these findings, maintaining vitamin D levels at or above 60.6 nmol/L may provide the greatest reduction in PRISm risk. However, levels above 50 nmol/L already reflect a significant reduction in risk, suggesting that public health strategies should prioritize addressing vitamin D deficiency (levels below 50 nmol/L) to mitigate the risk of PRISm effectively.

In conclusion, vitamin D may have a potential protective effect on PRISm. Preliminary explorations of its mechanisms suggest that vitamin D might play a role in the complex physiological and metabolic networks, particularly through mechanisms such as oxidative stress and insulin resistance. From a clinical perspective, physicians should consider incorporating the monitoring and management of vitamin D levels as part of a preventive strategy for populations at risk of PRISm and other related respiratory diseases. Including vitamin D assessments in routine clinical practice would help identify high-risk individuals and guide personalized interventions. Future intervention studies should focus on determining the optimal dose of vitamin D supplementation and its potential clinical applications in the prevention of PRISm.

### Limitations

This study revealed an important association between vitamin D levels and PRISm and explored the mediating roles of biomarkers such as SII, TyG, and bilirubin. However, there are certain limitations and shortcomings. Firstly, the cross-sectional design of the study limits the ability to infer causality; although significant associations were found, a causal relationship between vitamin D levels and PRISm cannot be established. Secondly, the study relied on self-reported data and laboratory measurements from the NHANES database, which may be subject to information bias or measurement errors, affecting the precision of the findings.

Regarding the mediation analysis, while SII, TyG, and bilirubin provided insights into the potential mechanisms linking vitamin D and PRISm, these indicators have their limitations. For instance, SII, as an inflammatory marker, may not fully capture the complexity of chronic inflammation and is susceptible to acute physiological fluctuations. The TyG index, though commonly used to assess insulin resistance, may vary in applicability across different populations and may not encompass all metabolic pathways. Although bilirubin showed a positive mediating effect, its levels are influenced by factors such as liver function and diet, which could lead to confounding. Additionally, mediation analysis relies on a series of model assumptions that may not entirely reflect the real conditions of complex biological systems, potentially impacting the robustness of the conclusions.

Despite efforts to adjust for confounders such as age, sex, race, and socioeconomic status, the potential impact of residual confounding cannot be fully excluded. Moreover, as the sample was primarily derived from the U.S. population, the generalizability of the findings to other regions or ethnic groups may be limited due to potential differences in genetic, environmental, and dietary factors that influence vitamin D metabolism and respiratory health. Additionally, the use of a single time-point measurement for vitamin D may not fully capture long-term status due to seasonal or temporal variability. Future research should adopt longitudinal designs and experimental studies, such as randomized clinical trials, to establish causal pathways, validate these findings across diverse populations, and further explore other potential mediators to ensure the robustness and broader applicability of the results.

## Data Availability

The original contributions presented in the study are included in the article/Supplementary material, further inquiries can be directed to the corresponding author.
